# Fistula between the rectal stump and left iliac artery

**DOI:** 10.1186/s12893-022-01776-9

**Published:** 2022-09-14

**Authors:** Mariam Alshammari, Jumana Fatani, Ohood Alotaibi, Khalid Alhajri, Khalil Terro

**Affiliations:** 1 Department of General Surgery, Specialized Medical Center, Riyadh, Saudi Arabia; 2 Consultant Breast and Endocrine Surgery, Department of General Surgery, Prince Sultan Military Medical City, Riyadh, Saudi Arabia; 3 Consultant Laparoscopic and General Surgery, Department of General Surgery, Specialized Medical Center, Riyadh, Saudi Arabia

**Keywords:** Fistula, Rectal stump, Left iliac artery

## Abstract

**Background:**

Isolated iliac artery aneurysms are rare and difficult to diagnose. It is more common in males. It can be asymptomatic at diagnosis or can present with frank rupture, or symptoms caused by compression on nearby organs.

**Case presentation:**

A 44 years old male was diagnosed with rectosigmoid adenocarcinoma and underwent low anterior resection. One year after the surgery, he presented with fistula between the rectal stump and left iliac artery that was managed by stenting.

**Conclusion:**

A fistula between the rectal stump and the left iliac artery is very rare. There are several treatment options for ilio-rectal fistula but no conclusive specific treatment.

## Background

One of the major causes of morbidity and mortality in the elderly is lower gastrointestinal bleeding (LGIB) with the diverticular disease being the most common cause in this population [1]. The mortality rate of LGIB is 2–4% [2]. A Massive LGIB from primary ilio-rectal fistula is rare and difficult to diagnose but it is more common than secondary fistulas resulting from previous vascular surgery [3]. Massive LGIB due to fistular communication between an internal iliac artery aneurysm and rectum is rarely seen in clinical practice. Immediate diagnosis and management determine the consequences [4]. In addition, the occurrence of a fistula between a false aneurysm and a rectal stump is similarly a rare phenomenon.

Herein we report a case of a patient with a fistula between the rectal stump and left iliac artery.

## Case presentation

### History and examination

A 44 years old male with no significant past medical or surgical history. Presented with a history of left lower abdominal pain, vomiting, flatulence and constipation for 2 months.

On examination, his abdomen was soft with left lower abdominal tenderness.

### Investigations

CBC and X-ray were unremarkable. An occult blood test was done and came out to be positive. Colonoscopy was performed and showed a lesion originating at 30 cm from the anal verge reaching the rectosigmoid junction. Sigmoid biopsy was obtained and was consistent with moderately differentiated invasive colonic adenocarcinoma.

A computerized tomography (CT) scan abdomen obtained with IV, oral and rectal contrasts revealed a diffuse and annular wall thickening of the sigmoid/descending colon in the left iliac fossa, this affected area was measuring up to about 68 mm in length and up to 10 mm in wall thickness, associated with diffuse adjacent fat stranding and tiny lymph nodes, narrowing its lumen without complete obstruction with adjacent 16 mm soft tissue density. Metastatic workup was negative.

### Management

The patient was referred to surgery and low anterior resection was done.

### Pathology

The pathology report was consistent with moderately differentiated invasive colonic adenocarcinoma of the rectosigmoid. The tumor size was 3.5 cm in greatest dimension and was invading through the muscularis propria into pericolic fat. All margins were free of cancer. There were intramural and extramural lymphovascular as well as perineural invasion. 1 out of 16 lymph nodes was positive. The pathologic stage was pT3N1M0.

### Follow up

The patient was started on adjuvant Capecitabine plus Oxaliplatin.

Upon further investigations for follow-up, positron emission tomography (PET) and CT scans showed metastasis to the lung and abdominal wall. He was referred for chemotherapy followed by radiotherapy to the lung lesions. The patient was then completed stereotactic radiosurgery (SRS) to the lung lesions. Post SRS, he was started on Avastin® (bevacizumab) every 3 weeks.

One year after the surgery, the patient presented to the emergency department (ER) unconscious. Upon thorough history, he was complaining of vomiting, diarrhea, lower left quadrant abdominal pain, and low-grade fever, 10 days before presenting to the ER. He was intubated and admitted to the intensive care unit (ICU) as a case of septic shock.

Head CT scan was unremarkable. CT showed diffusely thickened colon with pericolonic stranding, associated with the possibility of localized collection and abnormal air foci suggestive of localized infection adjacent to the anastomotic site with possible involvement of the small bowel.

After stabilization, the patient was taken for surgery. During laparotomy, there were extensive adhesions, a small bowel loop adhered to the site of the previous anastomosis. A moderate amount of free fluid was found. Features were in keeping with perforation with a contained leak. The small bowel loop was released from the old anastomosis, resection and anastomosis were done along with a diseased colon that was resected with adequate margin. End colostomy was created. He was then discharged in good condition.

The pathology report showed mucosal ulceration with thrombosed blood vessels and inflammatory cells infiltration.

2 weeks after the surgery, the patient presented to ER with massive bleeding per rectum, a portable ultrasound was done in the ER revealed that the rectosigmoid is distended with echogenic material that suggested blood. There were multiple pockets of free fluid in the abdomen, the largest is seen on the left measuring about 53 × 26 mm. Sigmoidoscopy was done, examining up to 16 cm from the anus, there were huge clots of blood with no visible source of bleeding. He was resuscitated with blood transfusion. An interventional radiologist was consulted. Selective crossover of left external iliac artery (EIA) small pseudoaneurysm and intimal irregularity with narrowing 2 cm segment in the proximal portion. That was covered with a stent. Post ballooning angiogram showed clear patency of the left EIA with a smooth outline of the wall, distal left lower limb angiogram showed mild spasm in the left superficial femoral artery (SFA). 4 days later he was discharged in good condition.

Unfortunately, the patient passed away after developing cerebellum brain metastasis. Which he underwent craniotomy for cerebellum mass resection that was positive for metastatic deposits of adenocarcinoma.

## Discussion

Aneurysms of the iliac artery can be classified into those related to abdominal aortic aneurysms, observed in 10–20% of cases and isolated aneurysms of the iliac artery [6]. Isolated iliac artery aneurysms are a very rare phenomenon accounting for 0.3–2% of all intra-abdominal aneurysms [6]. The most common site is the common iliac artery followed by the internal iliac artery with the external iliac artery being rarely involved [6]. It is predominantly seen in males with a ratio of 7:1 and it is more common in elderlies above 70.

Causes recognized to be associated with iliac artery aneurysms include atherosclerosis, mycotic infections, intraoperative or trauma injuries, collagen vascular diseases, and connective tissue diseases such as Marfan syndrome and Ehlers-Danlos syndrome. Pseudoaneurysms are caused by erosions resulting from malignancies [7, 8].

Formation of fistula can be noticed as a complication of low anterior resection (LAR), as blood vessels thinning and staple line tension with erosion that resulted from surgical manipulation [5]. In the present case, we hypothesize that previous LAR led to the formation of the fistula.

The natural history of these aneurysms is unclear however, the increase in the size of an aneurysm is associated with an increased risk of rupture. At diagnosis, the average size of an isolated internal iliac artery aneurysm (IIIAA) was 7.7 cm with a 33% incidence of rupture, although the overall mean size of ruptured aneurysms was 5.6 cm, as reported by Richardson and Greenfield. In another study by Brin and Busuttil, a rupture was seen in 38% with a 58% mortality rate although they could not find a direct relationship between the size of the aneurysm and the risk of rupture [9, 10].

IIIAA can be asymptomatic at diagnosis and can present with frank rupture, or symptoms elicited by compression on adjacent organs, the ureter anteriorly, the internal iliac vein and the lumbosacral trunk posteriorly, the external iliac vein and obturator nerve laterally, and the colon and small bowel medially [3]. A previous study reported 94 patients with 34 of them could not elicit the symptoms at presentation as they did not have enough information. Of the remained 60 cases, 13.3% were asymptomatic at diagnosis. Other presenting symptoms included abdominal pain in 31.7%, urinary symptoms or renal failure in 28.3%, lumbosacral pain in 18.3%, groin pain in 11.7%, hip or buttock pain in 8.3%, rectal bleeding or constipation in 8.3%, and deep vein thrombosis in 1.7% [3]. A rare case of a ruptured internal iliac aneurysm resulting in large bowel obstruction has also been reported [9]. Aneurysm rupture accounts for 40% of cases of isolated iliac artery aneurysms with an over 50% mortality rate [8].

An aneurysmal rupture is greatly indicated in case of iliac artery aneurysm with unstable vitals. Iliac artery aneurysms can be palpated as a pulsatile mass during digital rectal or vaginal examinations and they are more beneficial than abdominal examinations [9]. When rupture occurs, a scrotal and perianal hematoma can be seen [9].

The treatment of iliac aneurysms has considerably changed over recent years. The management of choice now in patients with an anatomically suitable and uncomplicated IIAA is endovascular treatment. Proximal ligation with obliterative endoaneurysmorrhaphy is one of the surgical managements in case of rupture or fistula as ligation alone is associated with an increased risk of recurrence [10]. Previously reported treatments for iliac artery to distal gastrointestinal tract fistula include Hartmann’s procedure, endovascular stenting, and vascular bypass [5].

**Fig. 1:**
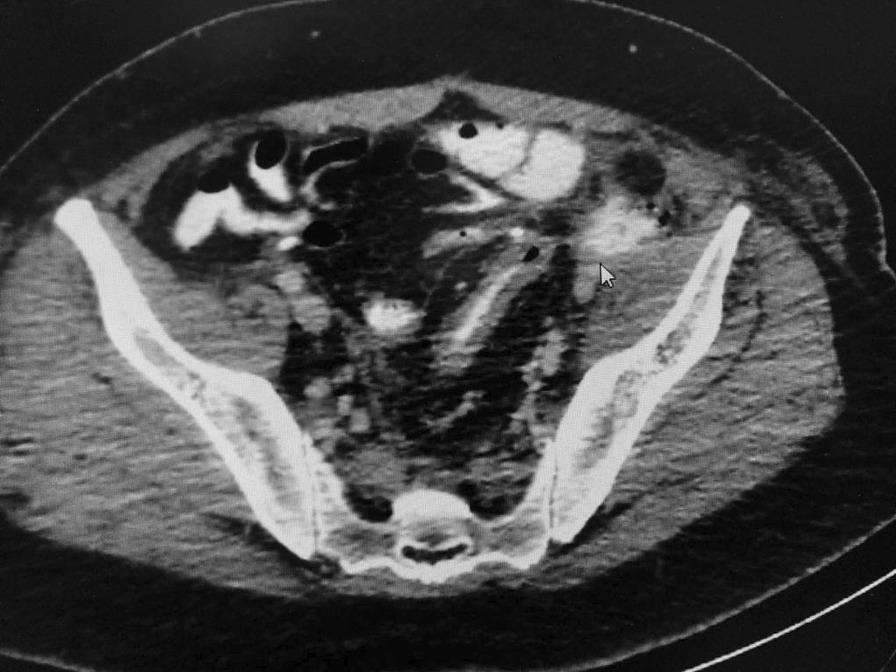
Sagittal CT showing fluid collection seen in the left iliac fossa anterior to the left external iliac artery

**Fig. 2:**
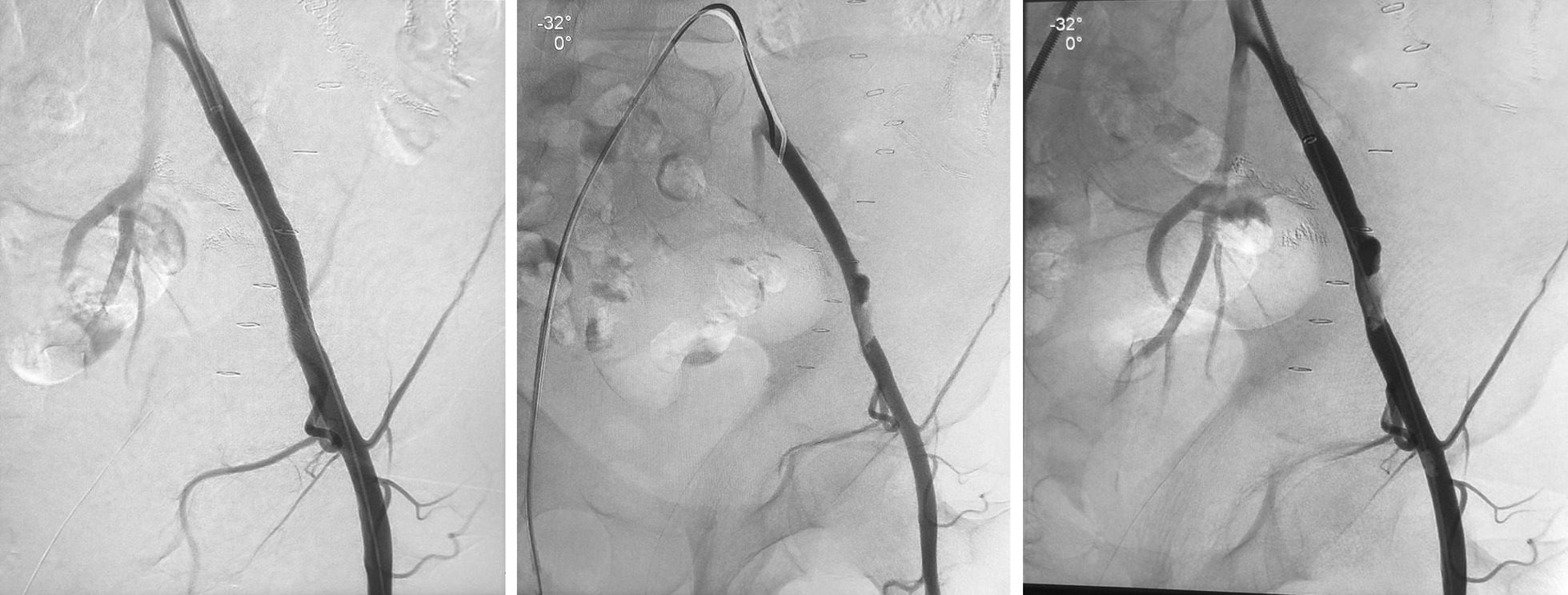
Lower limb angiogram showing stenting of left external iliac artery

In conclusion, a fistula between the rectal stump and the left iliac artery is very rare. Diagnosis is difficult and no specific investigation is available. Patients with massive LGIB with failure of an endoscope may need to consider imaging to rule out a fistula. There are several treatment options for ilio-rectal fistula but no conclusive specific treatment, it depends on the patient’s situation and physician’s experience. Endovascular or embolization is a minimally invasive surgery and is suitable for patients with high operative risk. It is a simple emergency and effective procedure that saves a patient’s life and can be performed by a general surgeon (Figs. 1 and 2).

## Data Availability

Our manuscript does not compromise anonymity or confidentiality of the patient.

## Abbreviations

LGIB: Lower gastrointestinal bleeding
CT: Computerized tomography
PET: Positron emission tomography
MSI: Microsatellite instability
SRS: Stereotactic radiosurgery
ER: Emergency department
ICU: Intensive care unit
EIA: External iliac artery
SFA: Superficial femoral artery
IIIAA: Isolated internal iliac artery aneurysm
